# Hemoglobin concentration; a pathway to frailty

**DOI:** 10.1186/s12877-020-01597-6

**Published:** 2020-06-11

**Authors:** Zara Steinmeyer, Cyrille Delpierre, Gaelle Soriano, Armand Steinmeyer, Loic Ysebaert, Laurent Balardy, Sandrine Sourdet

**Affiliations:** 1 Centre Hospitalier Universitaire de Toulouse, Geriatrics, Toulouse, France; 2 Institut National de la Santé et de la Recherche Médicale UMR 1027, Toulouse, France; 3 Institut Universitaire de Cancer de Toulouse, Toulouse, France

**Keywords:** Frailty-hemoglobin-Anemia

## Abstract

**Background:**

Frailty and hemoglobin concentration, above what would be considered clinical anemia, are two common findings in older patients that lead to an increased risk of negative health outcomes. The objective of this study is to evaluate whether hemoglobin concentration is an independent predictor of frailty and investigate possible causal pathways with a focus on the relationship between inflammation or nutrition and hemoglobin concentration.

**Methods:**

1829 community-dwelling participants aged 65 years or older who visited the Toulouse frailty day hospital during 2011 and 2016 were included in this analysis. Patients underwent a comprehensive geriatric assessment and had a blood sample taken.

A series of multivariate logistic regression models were performed after minimizing potential influence from age, gender, kidney function, inflammation, cognition, nutritional status and certain socio-economic factors.

**Results:**

Hemoglobin concentration and frailty are significantly associated after minimizing potential influence from other covariates (*p* < 0.005). An increase in one point of hemoglobin concentration is associated with a 14% risk reduction of being frail (OR = 0.86, 95%IC = 0.79–0.94). There was no evidence of a significant causal relationship between inflammation and nutritional status in the relationship between hemoglobin concentration and frailty status (*p* > 0.005).

**Conclusions:**

Hemoglobin concentration is strongly associated with frailty in older adults. These results can have potentially important implications for prevention policies targeting frailty by identifying potential patients with high risk of adverse outcomes and functional outcomes.

## Background

Frailty is a geriatric syndrome, prevalent in old age leading to an increased risk of negative health outcomes such as morbidity, mortality [1] and appears to be a transitional state in a dynamic process from robustness to disability [2]. It is defined as a state of increased vulnerability to stressors resulting from age-related decline in physiological reserve [3].

As a clinical condition, it is characterized by the presence of more than three of the five Fried criteria (intentional weight loss, self-reported exhaustion, weakness, slow walking speed and low physical activity [4]) and is based on the relationship between biological and age associated markers linked in a cycle of frailty.

With demographic changes in society such as population aging, prevention of disability has risen to the fore of medical and economic concerns. Despite research and publications, mechanisms of frailty development remain poorly understood [5]

Anemia and frailty are two common findings in geriatric patients and have been shown to be associated with similar poor health outcomes [6]. Anemia leads to diminished tissue oxygenation which may provoke diverse symptoms such as fatigue, weakness, and increased physical impairment. The symptoms of anemia are highly correlated with the symptoms associated to frailty and therefore may highlight a relationship between hemoglobin count and frailty [7, 8].

While the criteria as set out by the World Health Organization (WHO) are universally accepted, further study of anemia and frailty are frustrated by the continued debate of the cut off values chosen to define anemia in older populations [9, 10]. Certain studies have shown that lower hemoglobin count, above what would be considered clinical anemia predicts negative health outcomes such as mortality, morbidity and low physical performance [10–12]. As such there is growing evidence that lower hemoglobin concentration is associated to frailty given their high prevalence together in an older population and their common health outcomes [13]. Whether low hemoglobin results in a state of frailty or vice versa, or are the symptoms of a common physiological state is yet to be investigated.

The close relationship between lower hemoglobin concentration and the definition of frailty suggests that lower hemoglobin concentration could be a stepping stone in explaining the frailty syndrome. Cecchi et al. show in their study that lower hemoglobin concentration is associated with the decline of self reported physical activity and muscular strength which are potential correlates of frailty [11].

However, whether this association is independent from increased prevalence of comorbidity, causing both low hemoglobin and reduced physical function is under debate. There are a few studies to this day on physiological mechanisms between hemoglobin concentration and frailty [6, 13, 14]. Frailty may be caused by the influence of a range of variables: sociodemographic, physical, biological, lifestyle and psychological factors [15]. Certain of these variables share a common influence on hemoglobin concentration such as nutritional deficiency, chronic renal failure and chronic inflammation [16].

Identifying the respective role of these potential confounders is necessary to study the linkages that exist in the relationship between frailty and hemoglobin concentration.

An important remaining issue is to disentangle common and separate pathways by which both nutritional and inflammation mechanisms can influence this relationship [16, 17].

This study aims to examine the pathways linking hemoglobin concentration to the presence of a frailty syndrome. Our objective was to evaluate whether hemoglobin concentration was an independent predictor of frailty and investigate possibe causal pathways, in particuliar the relationship between inflammation and nutrition with hemoglobin concentration.

## Methods

### Participants

A cross-sectional study was conducted on community-dwelling participants aged 65 years or older who visited the Toulouse frailty clinic during 2011 and 2016. Each patient was referred by a physician (general practitioner, geriatrician or specialist) who had reported signs or symptoms of frailty using the Gérontopôle Frailty Screeening tool [18].

Patients who were referred by a physician came to the Toulouse frailty day hospital for a multidisciplinary evaluation. Socio-demographic, anthropometric, detailed medical history, functional, frailty status and disability was recorded, as well as blood sample collection.

Patients who underwent a comprehensive geriatric assessment and had a blood draw were assessed for eligibility.

Patients referred for an onco-geriatric evaluation were excluded from the study because they have an on-going inflammatory state (*N* = 419), as well as patients treated with erythropoietin (*N* = 7).

### Outcome variable

Frailty syndrome was evaluated according to the phenotype proposed by Fried et al. [4] based on the five criteria: unintentional weight loss, self-reported exhaustion, weakness, slow walking speed and low physical activity. Physical activity was the only adapted criterion as the Minnesota Leisure Time Questionnaire was not feasible in clinical practice. The questionnaire from the InChianti study on regular physical activity was used instead [19].

Weigth loss was defined as an unintentional loss of > 5 kg in the past year [20].

Exhaustion was present, if the participant answered often or most of the time for the question « How often in the last week did you feel that everything that you did was an effort? » used in the Center for epidemiologic studies-Depression scale [21].

Low physical activity was described as an absent to minimal activity level in the past year.

Slow walking speed was defined by gender and height specific cut-off values proposed by Fried over a 4 m course at usual pace.

Weakness was evaluated by hand grip strength measured by a handheld dynamometer (Jamar, Inrvington, NY) and based on gender, BMI specific cut-off values proposed by Fried [4]. Measures were done twice and on both hands, the average of the best results were used.

Following this evaluation, participants were considered frail if they had more than 3 criteria, the others were considered non frail.

### Main exposure

Blood tests were performed in the morning at enrollment in the frailty day clinic hospital. Samples were then sent and processed on automated instruments in the Toulouse University hospital laboratories. Hemoglobin concentration g/dl was measured using the hematology analyzer Sysmex spectrophometry using cyanmethemoglobin method.

### Covariates

Our study was designed to explore the relationship between frailty and hemoglobin count while controling for covariates that modify this relationship. This was done in order to see the pathways that exist in this relationship between frailty and hemoglobin concentration. Covariates likely to influence the main association tested between hemoglobin and frailty status were selected a pirori based on literature and added by order of influence. Covariates such as inherent demographics (age,sex) and health indicators were included. The other covariates were chosen based on their common association in the literature with frailty and hemoglobin concentration: kidney function, inflammation, cognition and nutritional status and socioeconomic positions [16, 22].

Two main types of covariates were distinguished:

#### Clinical variables

- Inflammation defined by a C-reactive protein level above 10 mg/dl [23]. Serum levels of high-sensitivity C-reactive protein (hs-CRP) is measured by immunoturbidimetry (Roche Cobas analyzer) [24].
- Renal function is estimated with glomerular filtration rate (GFR) calculated by using the chronic Kidney Disease Epidemiology Collaboration (CKD-EPI) equation [25]. Serum creatinine level was assessed using a Diazyme’s enzymatic method (Roche Cobas analyzer). The GFR categories were mapped to the categories retained by The Kidney Disease: Improving Global Outcomes (KDIGO) guidelines [26]. Normal kidney function was defined as a GFR ≥ 90 ml/min per 1.73m^2^, mildly decrease GFR between 60 and 89 ml/min per 1.73m^2^, moderate to severe decrease GFR 59-30 ml/min per 1.73m^2^ and severe decrease GFR > 29 ml/min per 1.73m^2^.
- The Mini nutritional assessment (MNA) was used to evaluate nutritionnal status [27]. A MNA score ≥ 24 indicated an adequate nutritional status, a MNA score < 17 a protein-calorie malnutrition and a MNA score between 17and 23.5 a risk of malnutrition.
- The Mini Mental State Examination (MMSE) as developed by Folstein was used as a surrogate for cognitive function [28, 29]. A MMSE score above 26 was considered as absence or questionable dementia, between 21 to 25 for mild, 11 to 20 as moderate and under 10 as severe dementia [30]

#### Social variables

To assess the subjects’s socio-economic position, we selected proxy variables such as the level of education. Education levels are categorized using the International Standard Classification of Education 2011 [31]. Education categories were defined as low (unschooled or primary education), medium (middle school to high school) or high (university level). We also collected living arrangements defined as either living alone or living with others (spouse, family,...).

### Statistical analysis

Sample characteristics were first described. Data are reported as percentage or as mean ± standard deviation.

We tested the normal distribution for the quantitative variables using the Shapiro wilk test.

Hemoglobin concentration was entered as a continuous measure as there was a linear relationship with frailty.

Bivariate analysis were used to assess the relationship between the covariates to hemoglobin and to frailty. Significance was tested using chi-square tests for categorical variables, Wilcoxon rank sum tests and Kruskall Wallis for continuous variables as appropriate.

Associations between age and hemoglobin were measured using the Pearson correlation coefficient.

To explore whether the increased risk of being frail was associated with hemoglobin concentration reflected the presence of comorbidity rather than constituing an additional risk factor of being frail, we further adjusted for kidney function, inflammation, cognition and nutritional status traditionnaly associated with frailty and hemoglobin concentration and we finally adjusted for socioeconomic position.

Multivariate logistic regression analysis with a forward selection was used to to examine the influence of these covariates further. Series of logistic regression models were performed.

Starting with the addition in the differents models of inherent individual covariates such as demographic data (Model 1), then biological parameters: renal function (Model 2), and inflammatory parameter (Model 3) and finally variables with environmental influence such as cognitive (Model 4), nutritional (Model 5) and socio economic variables (Model 6).

In Model 7 we added in our regression model the interaction of inflammation and nutritional status with hemoglobin concentration corresponding to the full model.

We determined the respective statistical contributions of confounders in explaining the association between hemoglobin concentration and frailty by using a traditional approach to mediation.

This analysis was conducted to investigate the possible combined effects of inflammation and nutrition on hemoglobin concentration.

The analysis was performed using STATA® version 11 (Stata Corp.,College Station, TX).

## Results

**Fig. 1:**
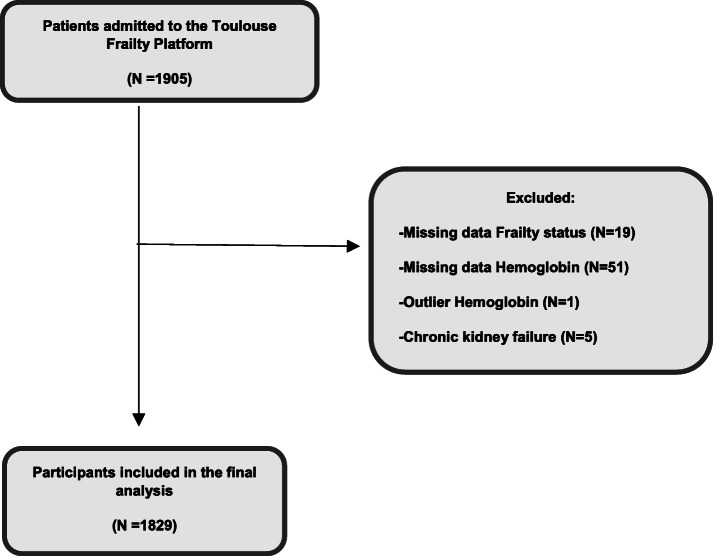
Flow chart showing sample selection

Patients who underwent comprehensive geriatric assessment and had a blood draw were assessed for eligibility (*n* = 1905). The flow chart corresponding to the sample selection used for this study is presented in Fig. 1. A total of 76 patients were excluded: 70 patients due to lack of data on hemoglobine or frailty status and 6 because they had aberrant biological results.

At baseline, the mean age of the participants included in the study was 82.4 years (±6.5), 32.64% were male, and 38.87% completed at least high school (Table 1).

**Table 1:** Baseline characteristics of the study subjects

Baseline Characteristic (***N*** = 1905)		
	Available data	Included (*N* = 1829)
**Socio-demographic**		
Age, mean (SD), years	1905	82.44 (6.50)
Gender, Male, n(%)	1905	597 (32.64)
School education, n(%)		
Low level of education	1811	677 (37.01)
Middle level of education		711 (38.87)
High level of education		357 (19.52)
Living arrangements n(%)		
Living alone	1874	842 (46.04)
Living with others		959 (52.43)
**Health conditions**		
Hypertension n(%)	1814	952 (52.05)
Diabetes mellitus n(%)	1814	261 (14.27)
Atrial arrhythmia n(%)	1811	278 (15.20)
Thrombo embolic disease n(%)	1808	141 (7.71)
Cardiovascular disease n(%)	1810	269 (14.71)
Congestive heart failure n(%)	1813	103 (5.63)
Chronic respiratory disease n(%)	1269	17 (0.93)
Renal insufficiency n(%)	1269	57 (3.12)
Connectivitis n(%)	1814	62 (3.39)
Prebaseline cancer n(%)	1814	337 (18.43)
**Examination findings**		
Frailty Fried’s criteria		
Non frail	1886	936 (51.18)
Frail		893 (48.82)
Cognitive: MMSE score (%)		
No impairment ≥26	1851	961 (52.54)
Mild impairment 21–25		484 (26.46)
Moderate impairment 11–20		310 (16.95)
Severe impairment 0–10		27 (1.48)
Nutritional status		
MNA score ≥ 24	1847	1084 (59.27)
MNA score 17–23.5		610 (33.35)
MNA score < 17		81 (4.43)
ADL score(/6), median (IQR)	1899	6 (1)
IADL score(/8), median (IQR)	1884	6 (4)
**Laboratory results**		
Hemoglobin, Mean ± SD g/dl	1854	13.33 (1.41)
Mean corpuscular volume, median (IQR), fl	1854	91 (6)
White blood cell count, median (IQR), 10^3/cm	1854	6.63 (2.38)
Platelets, median (IQR), 10^3/cm	1832	231 (80)
Anemia n(%)	1832	381 (20.83)
C-reactive protein, median (IQR), mg/L	1861	2.1 (3.9)
Renal function: CKD-EPI MD		
Normal (eGFR ≥90 ml/min/1.73m^2^), n(%)	1875	162 (8.86)
Mild decrease (eGFR 60–89 ml/min/1.73m^2^), n(%)		1120 (61.24)
Moderate to severe decrease (eGFR 30–59 ml/min/1.73m^2^), n(%)		484 (26.46)
Severe decrease (eGFR≤29 ml/min/1.73m^2^), n(%)		43 (2.35)

*SD* Standard deviation, *IQR* Inter quartile; MMSE: Mini mental state examination, *MNA* Mini nutritional assessment, *ADL* Activities of daily living, *IADL* Instrumental activities of daily living

Cardiovascular disease: Arrythmia, coronary artery disease, pulmonary hypertension, heart valve problems, congestive heart failure, hypertension, cholesterol, obliterating artery disease, thrombosis, pulmonary embolism;Cancer: leukemia, lymphoma, multiple myeloma, solid cancer

CKD-EPI MD: Chronic kidney disease epidemiology collaboration; eGFR: Equations for glomerular filtration rate

The most-common diseases was hypertension 52.05%. Most of the patients did not have organ insufficiencies nor history of inflammatory diseases and 18.43% of the subjects had a history of cancer. Hemoglobin concentrations ranged from 7.8 g/dl to 17.6 g/dl with a mean 13.33 g/dl (±1.41).

Based on the WHO criteria for anemia, 20.83% of participants were anemic at enrollement. 51.18% of the participants were non frail and were relatively autonomous with a mean score on the activities of daily living scale (ADL) of 5(±1) and on the instrumental activities of daily living scale (IADL) of 5(±2) [32].

Table 2 presents the results of multivariate logistic regression analysis conducted to study the relationship between hemoglobin and frailty.

**Table 2:** Odds of being Frail. Multivariate regression models (*N* = 1599)

	Model 1			Model 2			Model 3			Model 4			Model 5			Model 6		
	OR	CI 95%	p	OR	CI 95%	p	OR	CI 95%	p	OR	CI 95%	P	OR	CI 95%	p	OR	CI95%	p
**Age (years)**																		
< 79	1			1			1			1			1			1		
79–83	1.49	(1.11–2.00)	0.008*	1.53	(1.14–2.06)	0.005*	1.58	(1.17–2.14)	0.003*	1.52	(1.12–2.06)	0.007*	1.58	(1.15–2.18)	0.005*	1.56	(1.13–2.14)	0.007*
83–87	1.99	(1.48–2.65)	< 0.001*	2.03	(1.50–2.75)	< 0.001*	2.10	(1.55–2.85)	< 0.001*	1.84	(1.35–2.52)	< 0.001*	1.84	(1.33–2.55)	< 0.001*	1.86	(1.34–2.57)	< 0.001*
> 87	3.80	(2.80–5.12)	< 0.001*	3.85	(2.80–5.28)	< 0.001*	4.03	(2.92–5.56)	0.000*	3.45	(2.49–4.79)	0.000*	3.20	(2.27–4.49)	< 0.001*	3.23	(2.29–4.55)	< 0.001*
**Sex**																		
Male	1			1			1			1			1			1		
Female	1.41	(1.12–1.77)	0.004*	1.42	(1.13–1.79)	0.003*	1.48	(1.17–1.88)	0.001*	1.48	(1.16–1.88)	0.001*	1.44	(1.13–1.85)	0.004*	1.48	(1.14–1.92)	0.003*
**Hemoglobin count (g/dl)**	0.78	(0.72–0.85)	< 0.001*	0.79	(0.73–0.86)	< 0.001*	0.81	(0.75–0.89)	< 0.001*	0.82	(0.75–0.89)	< 0.001*	0.86	(0.79–0.94)	0.001*	0.86	(0.79–0.94)	0.001*
**Kidney function (eGFR)**																		
Normal				1			1			1			1			1		
Mild decrease				0.75	(0.51–1.10)	0.14	0.75	(0.51–1.11)	0.15	0.77	(0.52–1.15)	0.20	0.80	(0.53–1.21)	0.30	0.82	(0.54–1.25)	0.36
Moderate to severe decrease				0.82	(0.53–1.25)	0.36	0.81	(0.52–1.24)	0.33	0.84	(0.54–1.30)	0.43	0.88	(0.56–1.40)	0.60	0.90	(0.57–1.44)	0.67
Severe decrease				0.92	(0.40–2.08)	0.84	0.82	(0.36–1.87)	0.64	0.86	(0.37–1.99)	0.72	0.84	(0.35–2.00)	0.70	0.80	(0.34–1.91)	0.62
**Inflammation status (CRP)**																		
No inflammation							1			1			1			1		
Inflammation							2.25	(1.58–3.20)	< 0.001*	2.26	(1.58–3.25)	< 0.001*	2.11	(1.45–3.07)	< 0.001*	2.12	(1.45–3.09)	< 0.001*
**Cognitive (MMSE)**																		
No impairment										1			1			1		
Mild impairment										1.74	(1.36–2.22)	< 0.001*	1.53	(1.18–1.98)	0.001*	1.44	(1.10–1.87)	0.007*
Moderate impairment										2.27	(1.68–3.08)	< 0.001*	1 .81	(1.32–2.50)	< 0.001*	1.65	(1.18–2.31)	0.003*
Severe impairment										4.29	(1.60–11.51)	0.004*	1.98	(0.71–5.51)	0.19	1.72	(0.62–4.81)	0.30
**Nutritional status (MNA)**																		
Normal													1			1		
Risk of malnutrition													3.23	(2.53–4.11)	0.000*	3.24	(2.55–4.13)	< 0.001*
Malnutrition													8.21	(4.03–16.74)	0.000*	8.15	(4.00–16.60)	< 0.001*
**School education level**																		
Low level of education																1		
Middle level of education																0.91	(0.70–1.18)	0.47
High level of education																0.69	(0.50–0.95)	0.025*
**Living arrangements**																		
Living alone																1		
Living with others																0.80	(0.63–1.01)	0.06

*eGF*R Equations for glomerular filtration rate, *CRP* C-reactive protein, *MMSE* Mini mental state examination, *MNA* Mini nutritional assessment

* *P*<0.05

The crude odds ratio (OR) between hemoglobin and frailty was 0.78 (95%CI 0.72–0.85) This OR of being frail decreased by 22% for an increase of hemoglobin of 1 g/dl after adjustment for gender and age (model 1), as after inclusion of kidney function (OR = 0.79, 95%CI = 0.73–0.86) (model 2). This link persisted after the inclusion in the model of inflammation (OR = 0.81, 95%CI = 0.75–0.89) (model 3), cognition (OR = 0.82, 95%CI = 0.75–0.89) (model 4), nutritional status (OR = 0.86, 95%CI = 0.79–0.94) (model 5) and socio economoic variables (OR = 0.86, 95%CI = 0.79–0.94) (model 6).

Regarding the other determinants of frailty, the risk of being frail increased according to the stage of kidney disease, but this association was not statistically significative.

Inflammation was associated with a 125% risk of being frail (model 3) and this link persisted after inclusion of cognition (OR = 2.26, 95%CI = 1.58–3.25) (model 4) as with the addition of nutritional status (OR = 2.11, 95%CI = 1.45–3.07) (model 5). The risk of being frail increased according to the degree of severity of the cognitive state (model 4). For example the risk of being frail was 4.29 times more higher in the severly impaired (OR = 4.29, 95%CI = 1.60–11.51). This risk was significantly attenuated once adding nutritional status (OR = 1.98, 95%CI = 0.71–5.51) and socio economic variables (OR = 1.72, 95%CI = 0.62–4.81).

Nutritional status was independantly associated with being frail (model 5). The risk of being frail was multiplied by 8 if the patient was in a state of malnutrition.

Education level was independently associated with being frail, the higher the level of education was, decreased the risk of being frail (model 6).

We found neither interaction between hemoglobin and nutrition to frailty, nor between hemoglobin and inflammation (*p* > 0.05).

## Discussion

This study examines the influence of hemoglobin concentration on the subsequent risk of being frail after adjustment for a large range of confounders.

By this approach, many confounding factors that may explain pathways between hemoglobin and frailty were examined. The main finding of our study is that we have identified a significant association between hemoglobin count and frailty in this community dwelling population (*p* < 0.005). An increase in one point of hemoglobin concentration is associated with a 14% risk decrease of being frail (OR = 0.86 IC95% 0.79–0.94).

This association remained significant after adjusting for age, gender, kidney function, inflammation, cognition, nutritional status and socio-economic position. The results of this study confirm results found in Corona et al. and Juarez-Cedillo et al.’s study but with a wider and older population sample [6, 13]. Moreover, the decision to expand the number of variables under consideration in this study to include kidney function, inflammation, cognition, nutritional status and socio economic variables as they have been identified as potential confounders to frailty and hemoglobin concentration allows a more thorough understanding of this relationship. Thus, they should be adapted in future studies to further assess the causal relationship between these factors.

Looking at the relationship between hemoglobin concentration and disability, Chaves and al. have questionned the clinical appropriateness of the definition of anemia set by the WHO hemoglobin level < 12 g/l for women and < 13 g/l for men [33] given the independent adverse effects of low hemoglobin on mobility function [34]

This seems to be confirmed by the independent association between hemoglobin concentration and frailty status found in our study. Indeed low hemoglobin puts older adults at risk of poor oxygen delivery with exhaustion, fatigue and failing muscle strength [33, 35] Symptoms which are each individual criteria in the definition of frailty by Fried [4].

This hypothesis highlights another issue, which are the mechanisms at stake in lowering hemoglobin concentration.

This was our second objective, studying potential confounders between hemoglobin and frailty. Among all these factors identified: kidney function, inflammation, cognition, nutritional status and socio-economic factors there was no impact on the effect of hemoglobin on frailty after adjustment. This suggests that the relationship between hemoglobin concentration and frailty is independent of these variables and that further studies are required to identify the potential links between them.

Our hypothesis was that lower hemoglobin concentration and frailty are associated to a state of chronic inflammation. Indeed, frail older adults have often a poorer health status, chronic conditions and comorbidites leading to an active low grade of inflammation lowering hemoglobin concentration [36, 37]. However, Walston et al. have provided evidence supporting an activation of inflammation with and without clinical comorbidities, suggesting that there is a direct relationship between frailty and inflammation [36]. Many studies have explored multiple inflammatory biomediators as potential mechanisms by which inflammation might promote lower hemoglobin [17, 38].

We chose in our study CRP as an inflammatory biomediator, after adding CRP in model 3 there was no impact on hemoglobin’s odds ratio (OR = 0.81, IC95%0.75–0.89 *p* < 0.005) and after studying the interaction between those two parameters the association was not significative (*p* = 0.14). The lack of relationship might be due to the cut off values used to define inflammation, or the inflammatory biomediator chosen [37, 39]. The pathogenesis of frailty and the role of inflammation remains incompletely understood. Leng et al.’s studied the potential role of neutrophils and monocytes in the pathogenesis of frailty as well as the role of interleukin-6 [14, 39]. There is a probable link between inflammation and frailty, our hypothesis was that maybe lower hemoglobin reflected this effect and could be a mediating factor we could follow to study this relationship. The question is if hemoglobin is a stigma of this relationship between inflammation and frailty or is it an independent actor in the frailty process.

We also studied the relationship between hemoglobin and nutritional status. Low hemoglobin count could be attributed to nutrient deficiencies (Iron, B12 and folate) [16], we kept in light this information and used the MNA [27] score as a proxy of the patient’s nutritional state. Indeed, a patient with malnutrition is more likely to have nutrient deficiencies.

In our study, the risk of being frail increases according to their nutritional status in a bivariate analysis. However, the interaction between frailty, hemoglobine and nutritional status was not significative in this study.

The absence of link between nutritional status and hemoglobine is possibly explained by two hypotheses.

One hypothesis is that nutritional status as determined by the MNA score does not define vitamin deficiency, as patients with vitamin deficiencies are not necessarily malnourished and vice versa [40]. Another hypothesis is that the MNA is a certain type of nutrition screening tool and thus might not identify all situations of malnutrition in the elderly [41].

The main strength of this study is that data was prospectively measured with a large number of participants who underwent a comprehensive frailty assessment and that we used an explanatory model with series of logistic regression taking into account numerous potential confounders.

There are also a number of limitations that need to be considered.

Firstly, we took in account the major confounding factors of hemoglobin concentration, we did not investigate thouroughly all the causes of low hemoglobin concentration as this would have exceeded the purpose of our study.

Secondly, this is a monocentric study, all the study participants have been recruited through the Toulouse frailty day hospital. This may induce a selection and a representativeness bias.

Moreover, participants were referred by a physician (general practitioner, geriatrician or specialist) who had reported signs or symptoms of using the gerontopôle frailty screening tool (GFST) [18], so our findings might be difficultly extended to a general community-dwelling elderly population.

Finally, the cross-sectional nature of our study is the main limitation, a longitudinal analysis may shed light on the chronological and possible etiological relationship between hemoglobin and frailty.

## Conclusions

Hemoglobin concentration is strongly associated with frailty in older adults. Addition of different covariates did not influence this relationship and the mechanisms involved in this relationship remain unclear. If hemoglobin concentration is an independent and a causal factor of frailty, improving concentrations of hemoglobin could potentially diminish the risk of frailty and thus detecting low hemoglobin above the score of anemia would be relevant.

Potential pathways linking hemoglobin to frailty deserve further investigations. These results can have potentially important implications for prevention policies targeting frailty, by identifying potential patients with high risk of adverse outcomes and functional outcomes.

## Data Availability

The datasets used and/or analysed during the current study are available from the corresponding author on reasonable request.

## Abbreviations

ADL: Activities of Daily Living
BMI: Body Mass Index
CI: Confidence Interval
CKD-EPI: Chronic Kidney Disease Epidemiology Collaboration
CRP: C-Reactive Protein
GFR: Glomerular Filtration Rate
GFST: Gerontopôle Frailty Screening Tool
IADL: Instrumental Activities of Daily Living
KDIGO: The Kidney Disease Improving Global Outcomes guidelines
MMSE: Mini Mental State Examination
MNA: Mini nutritional assessment
OR: Odds Ratio
WHO: World Health Organization
